# Expression and aberrant promoter methylation of Wnt inhibitory factor-1 in human astrocytomas

**DOI:** 10.1186/1756-9966-29-26

**Published:** 2010-03-24

**Authors:** Zhuanyi Yang, Ying Wang, Jiasheng Fang, Fenghua Chen, Jinfang Liu, Jun Wu, Yanjin Wang

**Affiliations:** 1 Department of Neurosurgery, Xiangya Hospital of Central South University, Changsha, Hunan, 410078, PR China; 2 Department of Pathology, Basic Medical School of Central South University, Changsha Hunan, 410078, PR China

## Abstract

**Background:**

Wnt inhibitory factor-1(WIF-1) acts as a Wnt-antagonists and tumor suppressor, but hypermethylation of WIF-1 gene promoter and low expression activate Wnt signaling aberrantly and induce the development of various human tumors. With this work we intended to investigate the expression and promoter methylation status of WIF-1 gene in human astrocytomas.

**Methods:**

The tissue samples consisted of 53 astrocytomas and 6 normal brain tissues. The expression levels of WIF-1 were determined by immunohistochemistry and semiquantitative RT-PCR. The results were analyzed in correlation with clinicopathological data. Methylation status of WIF-1 gene promoter was investigated using methylation specific PCR. The relationship between methylation and expression of the genes was analyzed.

**Results:**

The average expression levels of WIF-1 protein and mRNA in astrocytomas were decreased significantly compared with normal control tissues. The protein and mRNA expression of WIF-1 gene in astrocytomas was decreased with the increase of pathological grade. Furthermore, WIF-1 promoter methylation was observed by MS-PCR in astrocytomas which showed significant reduction of WIF-1 expression. The WIF-1 promoter hypermethylation was associated with reduced expression of WIF-1 expression.

**Conclusion:**

Our results demonstrate that the WIF-1 gene is frequently down-regulated or silenced in astrocytomas by aberrant promoter methylation. This may be an important mechanism in astrocytoma carcinogenesis.

## Background

Astrocytomas are the most common primary tumors of the central nervous system. Despite recent advances in diagnosis and therapies such as surgery, radiation, and chemotherapy, the prognosis and survival times of high-grade astrocytomas(WHO grade III, IV)remains poor. The median survival is only 12 to 15 months for patients with glioblastoma(WHO grade IV)and 2 to 5 years for patients with anaplastic astrocytoma(WHO grade III)[1].

The Wnt/β-catenin signaling pathway plays a significant role in various processes of early development and the pathogenesis of human diseases, including human malignancies. Recently, there are several reports which evident the involvement of Wnt/β-catenin signaling in astrocytomas [2-5]. Wnt inhibitory factor-1 (WIF-1) is identified as one of the secreted antagonists that can directly bind to Wnt proteins to inhibit Wnt/β-catenin signaling[6]. Down-regulation and promoter hypermethylation of WIF-1 gene have been reported in human hepatocellular, nasopharyngeal, pulmonary, urocystic and gastrointestinal malignancies [7-11]. Yet little is known regarding the expression and promoter methylation of WIF-1 in astrocytomas. In this study, we describe for the first time that the expression of WIF-1 was frequently downregulated by its promoter hypermethylation in astrocytomas compared with normal tissue samples, which might contribute to the upregulation of Wnt/β-catenin signaling in astrocytoma carcinogenesis.

## Materials and methods

### Patients and tissue samples

53 fresh astrocytoma samples (T1-T53)were collected after informed consent from patients who underwent brain operations for astrocytoma at Xiangya Hospital (Hunan, China). Immediately after surgical resection, portions of the tumors were frozen and stored at -80°C for RNA and DNA extraction, and the remanets were fixed in 10% formalin. Tumors were graded and classified according to the World Health Organization (2007)[12], including gradeI(1), grade II(22), grade III(12), and grade IV(18). In all cases of astrocytomas, there were 32 (60.38%) males and 21 (39.62%) females with the median age of 38.5 years (range: 5~66 years). For comparison, 6 normal human tissues (N1-N6) from patients with contusion and laceration of brain were obtained at the time of decompressive operation.

### Immunohistochemistry

WIF-1 protein expression was determined by using immunohistochemical staining (IHC) on formalin-fixed paraffin-embedded tissue sections. Briefly, 5 μm thick sections were deparaffinized, rehydrated using xylene and alcohol, incubated with 0.3% H_2_O_2 _to block endogenous peroxidase activity, and incubated with normal goat serum to block nonspecific antibody binding. Before immunostaining, antigen retrieval was done by immersing sections in a 10 mM concentration of citrate buffer (pH6.0) and boiling in a pressure cooker for 2 minutes. The sections were incubated at 4°C overnight with a 8 μg/ml monoclonal antibody against human WIF-1 protein (R&D, Minneapolis, USA). The sections were then incubated with biotinylated goat anti-mouse IgG antibody (Zymed, San Francisco, CA, USA) for 30 min. The antigen-antibody complexes were visualized using streptavidin-horseradish peroxidase conjugate (Zymed, San Francisco, CA, USA) and diaminobenzidine (DAB)as a chromogen.

The slides were counterstained with hematoxylin. For WIF-1 protein expression, nuclear staining was considered to be negative, whereas cytoplasmic and membranous expression was analyzed according to the intensity and proportion of positive cells to all cells[10]. IPP6.0 (Media Cybernetics, Bethesda, MD, USA)was applied to semiquantify immunohistochemical results. Staining was scored for intensity [0 (negative), 1+ (weak), and 2+ (strong)] and percentage of postive staining in malignant cells [0 (0-4%), 1 (5-24%),2 (25-49%), 3 (50-74%), or 4 (75-100%)]. The multiplication of intensity and percentage counts was used as the final immunohistochemistry scores [13]. For heterogenous staining patterns, each component was scored independently and the results were summed. For example, a specimen containing 25% tumor cells with strong intensity (1 × 2 + = 2), 25% tumor cells with weak intensity (1 × 1 + = 1), and 50% tumor cells without immnoreactivity received a score of 2 + 1 + 0 = 3. Cytoplasmic and membranous staining in normal brain tissue served as internal positive controls. Negative controls were included in the IHC analyses by omitting the primary antibody.

### RNA extraction and Semiquantitative RT-PCR

Total RNA from tumor tissues and normal tissues were isolated using a TRIzol procedure(Invitrogen, Carlsbuel, CA, USA). An equal amount of RNA from each sample was added to 25 μl of reaction mixture and cDNA was synthesized by First Strand cDNA Synthesis kit (Fermentas, Burlington, Canada). Primers for semiquantitative RT-PCR were obtained from Takaro (Dalian, China). Primer sequences for the human WIF-1 cDNA were 5'-CCGAAATGGAGGCTTTTGTA-3' (forward) and 5'-TGGTTGAGCAGTTTGCTTTG-3' (reverse)[8]. Glyceraldehyde-3-phosphate dehydrogenase (GAPDH) was used as an internal control. Primer sequences for GAPDH were 5'-CAATGACCCCTTCATTGACC-3' (forward) and 5'-TGGAAGATGGTGATGGGATT-3' (reverse). The cycle was defined at 95°C for 5 min, followed by 32 cycles of denaturing at 95°C for 30 sec, annealing at 56°C for 40 sec and extension at 72°C for 40 sec. This was followed by the final extension at 72°C for 10 min. The PCR products were electrophoresed in 2% agarose gels. Relative WIF-1 mRNA levels were evaluated by UVP software (UVP Inc., Upland, CA, USA) and were expressed as the fold-difference relative to GAPDH mRNA levels.

### Genomic DNA Extraction and Methylation-specific PCR (MS-PCR)

DNA was extracted from astrocytoma tissues by standard proteinase K digestion, phenol chloroform and ethanol precipitation proceeded. Bisulfite modification of genomic DNA was carried out by using a EZ DNA methylation kit (Zymo, CA, USA), according to the manufacturer's protocol. WIF-1 promoter region has been identified and described previously [14]. Bisulfite-treated genomic DNA was amplified using either a methylation-specific or an unmethylation-specific primer set. GC Rich DNA polymerase (Qiagen, Hilden, Germany) was used in the experiments. Sequences of the methylation-specific primers were 5'-GGGCGTTTTATTGGGCGTAT-3' (forward) and 5'-AAACCAACAATCAACGAAC-3' (reverse). Sequences of the unmethylation-specific primers were 5'-GGGTGTTTTATTGGGTGTAT-3' (forward) and 5'-AAACCAACAATCAACAAAAC-3' (reverse) corresponding to the WIF-1 promoter region sequences -488 to -468 and -310 to -290, respectively. The PCR was carried out in a Techne TC-412 Thermal Cycler(Keison, Essex, UK) under the following conditions: one cycle of 95°C for 10 min, followed by 35 cycles of denaturing at 94°C for 1 min, annealing at 60°C for 50 sec and extension at 72°C for 50 sec. This was followed by the final extension at 72°C for 10 min. The PCR products were analysed by electrophoresis on 2% agarose gel and samples were evaluated. Normal human lymphocyte DNA was either treated directly with sodium bisulfite or after in vitro methylation by SssI methyltransferase(New England Biolabs, Ipswich, MA) to serve as unmethylated and methylated controls, respectively.

### Statistical analysis

Statistical analyses were performed using SPSS software version 13.0(SPSS, Chicago, USA). Data were presented as mean ± SD. Differences of the variables between groups were tested by Student's *t *test. *P *< 0.05 was regarded as statistically significant for all the tests.

## Results

### Expression of WIF-1 protein

**Figure 1:**
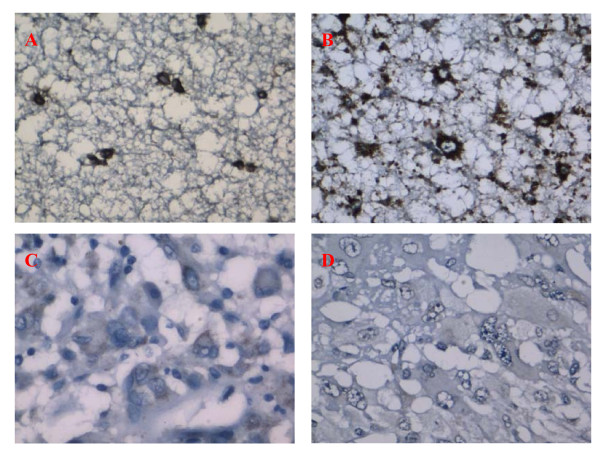
**Selected results of immunohistochemical analysis for anti-human WIF-1 antibodies**. Paraffin-embedded sections of representative astrocytomas and normal brain tissues were stained with the antibodies against human WIF-1. The products of WIF-1 expression (brown) located in cytoplasm and membrane. The photographs of A and B are normal brain tissues and pilocytic astrocytoma (WHO grade I)which showed strong staining for WIF-1, respectively. In contrast, the anaplastic astrocytoma(WHO grade III) and glioblastomas(WHO grade IV)that have weak or negative expression levels of WIF-1 were shown in C and D, respectively. Pathological malignancy grade of astrocytoma correlated with IHC score of WIF-1.

To detect the expression level of WIF-1, immunohistochemistry was performed in 6 normal brain tissues and in 53 astrocytoma tissues (Tab. 1 and Fig. 1). Reactivity was generally cytoplasmic and membranous. The average values of WIF-1 expression were 7.33 ± 0.52 and 2.94 ± 2.19 respectively in normal brain tissues and astrocytomas. Statistical analysis indicated that the level of WIF-1 expression was significantly lower in tumors than that in normal brain tissues (*P *< 0.001), and it was decreased as the pathological grade increased (*P *= 0.002) (Tab. 2). No significant correlation was found between WIF-1 protein expression and age(*P *= 0.53)or sex(*P *= 0.69)respectively.

**Table 1:** Patient's clinical data and results of our study

Sample	Sex	Age	WHO grade	IHC scores	mRNA	Methylation status
N1	F	60		7	0.927	U
N2	F	56		7	0.907	U
N3	M	28		7	0.862	U
N4	M	56		8	0.976	U
N5	F	27		8	0.915	U
N6	M	57		7	0.791	U
T1	M	43	II	2	0.107	U/M
T2	F	50	III	0	0	M
T3	F	38	II	5	0.653	U
T4	M	34	III	0	0	M
T5	F	57	II	2	0.658	U
T6	M	61	III	5	0.773	U
T7	M	54	IV	5	0.602	U/M
T8	M	66	IV	1	0	M
T9	F	14	I	7	0.809	U
T10	F	40	II	2	0.151	M
T11	M	37	II	5	0.462	U
T12	M	43	II	3	0.769	U
T13	F	53	II	5	0.398	U
T14	M	27	II	5	0.716	U
T15	M	45	II	5	0.722	U
T16	F	45	IV	1	0.115	M
T17	F	39	III	6	0.897	U
T18	M	30	II	3	0.215	M
T19	M	40	IV	0	0.000	M
T20	F	33	II	5	0.704	U
T21	F	38	IV	0	0.000	M
T22	M	5	II	7	0.907	U
T23	M	51	IV	1	0.000	M
T24	M	66	II	5	0.478	U
T25	F	46	II	5	0.447	U
T26	M	55	III	1	0.134	U/M
T27	M	41	III	1	0.153	U/M
T28	M	43	IV	2	0.153	M
T29	F	39	IV	1	0.129	M
T30	M	29	IV	5	0.347	U
T31	M	16	IV	0	0.000	M
T32	F	55	IV	1	0.147	M
T33	M	58	IV	2	0.189	U/M
T34	F	27	IV	1	0.131	M
T35	M	58	IV	1	0.182	M
T36	M	50	IV	3	0.122	M
T37	M	14	IV	2	0.337	U/M
T38	F	9	IV	4	0.334	U
T39	M	33	III	3	0.247	U/M
T40	F	19	III	5	0.783	U
T41	M	33	II	1	0.179	M
T42	M	38	II	2	0.164	M
T43	M	63	II	1	0.293	U/M
T44	F	37	III	2	0.158	M
T45	F	11	III	0	0.000	M
T46	M	27	III	5	0.523	U
T47	F	23	IV	3	0.467	U
T48	M	27	II	0	0.176	U/M
T49	F	28	II	6	0.828	U
T50	M	25	II	2	0.332	U
T51	M	40	II	8	0.903	U
T52	M	38	II	5	0.443	U
T53	F	48	III	4	0.324	U

N, normal brain tissue; T, astrocytoma tissue ;M, male; F, female; IHC, immunohistochemistry; U, unmethylation; M, methylation

**Table 2:** The relationship between the expression of WIF-1 and clinicopathological features in 53 cases of astrocytoma

Clinical signs	Number of Cases	IHC		RT-PCR	
			
		Scores	*P*-Value	QT	*P*-Value
**age**					
<39	26	3.23 ± 2.32	0.35	0.40 ± 0.30	0.23
≥39	27	2.67 ± 2.06		0.31 ± 0.27	
**sex**					
male	32	2.84 ± 2.17	0.69	0.33 ± 0.28	0.50
female	21	3.10 ± 2.26		0.38 ± 0.31	
**Pathological Grading**					
Low grade(I - II)	23	3.96 ± 2.16	0.002^a^	0.50 ± 0.27	0.001^b^
High grade(III - IV)	30	2.17 ± 1.90		0.24 ± 0.25	

^a, b ^Statistically significant (p < 0.05). QT, relative mRNA level of WIF-1.

### Expression of WIF-1 mRNA transcript

**Figure 2:**
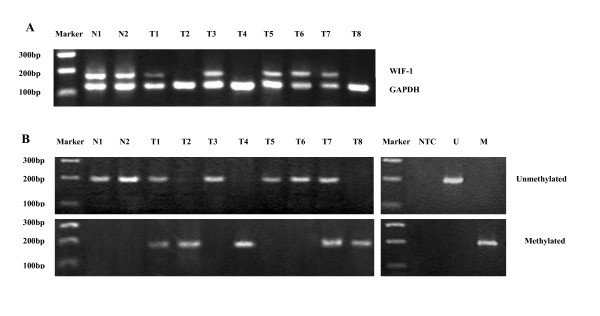
**Selected results of mRNA expression and promoter methylation of WIF-1**. A, RT-PCR results of the WIF-1 gene in normal brain tissue (N1-N2) and astrocytoma (T1-T8) is shown. GAPDH is shown as a control. The fragments of amplified human WIF-1 and GAPDH cDNA are188 and135 bp, respectively. B, Representative methylation status of the WIF-1 promoter in 10 matched pairs of normal brain tissue (N1-N2) and astrocytomas(T1-T8).T1,3,5:WHO grade II;T2,4,6:WHO grade III;T7,8: WHO grade IV. U, unmethylated control;M, methylated control;NTC, no template control.

Semiquantitative RT-PCR assay was also performed to analyze the expression of WIF-1 at transcription level. WIF-1 mRNA was examined in 6 normal brain tissues as well as in 53 resected astrocytoma tissues [Tab. 1 and Fig. 2(A)]. The results showed that WIF-1 expression in tumor samples (0.35 ± 0.29)was significantly lower compared with normal brain tissues (0.90 ± 0.06, *P *< 0.001). Significant association was found between WIF-1 mRNA downregulation and the pathological grade(*P*= 0.001). However, WIF-1 gene expression was not correlated with age(*P *= 0.23)or sex(*P *= 0.50) in tumor samples (Tab. 2).

### Relationship between promoter methylation and expression of WIF-1

To examine whether the methylation status of promoter correlates with the expression of WIF-1, MS-PCR was carried out [Tab. 1 and Fig. 2(B)]. No hypermethylation was obseved in all normal brain tissues. In contrast, aberrant methylation was observed in 29(54.72%) of 53 tumor samples. Especially, 22 (73.33%) of 30 high-grade astrocytomas(WHO grade III, IV) showed promoter hypermethylation.

**Figure 3:**
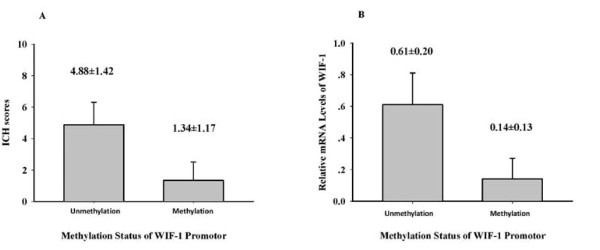
**Correlation between hypermethylation and decreased or weak expression of WIF-1 in astrocytomas**. A significant downregulation of the protein(A) and mRNA(B) expression of WIF-1 was observed in astrocytomas with promoter methylation(both *P *< 0.001). The bars in the graph showed the mean ± SD.

Unmethylation-specific PCR band was detected in 9 of 29(31.03%) methylated samples, probably due to unavoidable contamination of non-tumor cells, or partial methylation of the gene. The promoter methylated tumors showed low WIF-1 protein and mRNA expression, whereas the promoter unmethylated tumors displayed high protein and mRNA expression levels (Fig. 3). Thus, these data indicated a significant correlation (both *P *< 0.001) between hypermethylation and decreased expression of WIF-1 in astrocytomas.

## Discussion

WNT/β-catenin signaling pathway is important in tumorigenesis and embryogenesis [15,16]. The signaling pathway mediated by Wnt proteins currently includes two classes - canonical and noncanonical - on the basis of the activity of Wnt proteins in cell lines or in vivo assays. The canonical pathway, in which β-Catenin plays a crucial role, is the most studied Wnt pathway in cancers. The activation of canonical pathway allows β-catenin to accumulate in the cytosol and enter the nucleus and induces expression of Wnt target genes like c-Myc, N-Myc, and cyclin D1 [17-19], many of which have been implicaticated in human cancers. In astrocytoma, the level of Wnt-2, Wnt-5a and β-catenin protein is strikingly increased compared with normal brain tissue[2,3,5]. Knockdown of Wnt and its key mediator β-catenin in the canonical Wnt pathway by siRNA in human astrocytoma cells inhibited cell proliferation and invasive ability and induced apoptotic cell death, and reduced tumorigenicity in vivo. The above findings suggest Wnt signaling in astrocytoma is constitutively activated and of critical importance in the astrocytoma genesis.

WIF-1 is an endogenous Wnt antagonist. Downregulation of WIF-1 may release the inhibitory effect exerted by WIF-1 on the WNT/β-catenin signaling[20]. This then enhances the accumulation of β-catenin and promotes tumorigenesis. Although it is known that WIF-1 is strongly expressed in embryonic mouse brain [21], its expression in brain tumors has not yet been a matter of investigation. In this study, we analysed the protein and mRNA level of WIF-1 in astrocytomas using immunohistochemistry and RT-PCR. The level of protein and mRNA expression in astrocytomas was significantly lower than that in normal tissues. As the pathological grade increased, the protein and mRNA expression of WIF-1 gene in astrocytoma were decreased. These results indicated that WIF-1 was frequently and significantly downregulated in astrocytomas, especially in high-grade astrocytomas, which might contribute to the upregulation of Wnt/β-catenin signaling in astrocytoma carcinogenesis.

Aberrant methylation of promoter regions that silences transcription of the genes has been recognized as a mechanism for inactivating tumor suppressor genes in human cancer [22,23]. It occurs at cytosine bases located 5' to a guanosine and so-called CpG dinucleotide short regions of CpG dinucleotides known as CpG islands are found in the proximal promoter region of over half of human genes [23]. The methylation of these gene promoters is generally not detected in normal tissues but in the hypermethylation of CpG islands resulting in a loss of gene function, which is a common feature in many tumor types. Now, many other genes such as LHX9, MGMT, CDKN2A, PTEN, and P15 have been shown to be methylated in astrocytomas [24-28]. WIF-1 silencing may be an early epigenetically carcinogenic event and plays a role in tumor development and progression[29]. In this study, we demonstrated that WIF-1 downregulation or silencing was associated with aberrant methylation of promoter region in malignant astrocytoma tissue samples. This finding reveals an important epigenetic event during the development of astrocytoma, suggesting that WIF-1 may be a key antagonist of Wnt signaling in astrocytoma.

In summary, we provide evidence that WIF-1 is not only frequently hypermethylated in astrocytomas but this epigenetic alteration of the WIF-1 gene is associated with reduced expression. This study reveals a novel epigenetic event in the pathogenesis of astrocytoma, which may shed light on developing new approaches for this fatal disease. The reversibility of methylation silencing may allow restoration of WIF-1 function and regulation of Wnt signaling. This could be important in the development of new and effective strategy in astrocytoma treatment.

## Competing interests

The authors declare that they have no competing interests.

## Authors' contributions

YZY and YW carried out the experiment of this manuscript and drafted the manuscript. YZY and JSF participated in the design of the study and organized the whole study process. FHC, JFL and JW participated the experiment and revised the manuscript. YZY and YJW conceived the study project, provided financial support. All authors read and approved the final manuscript.
